# DNA accumulation on ventilation system filters in university buildings in Singapore

**DOI:** 10.1371/journal.pone.0186295

**Published:** 2017-10-12

**Authors:** Irvan Luhung, Yan Wu, Siyu Xu, Naomichi Yamamoto, Victor Wei-Chung Chang, William W. Nazaroff

**Affiliations:** 1 Berkeley Education Alliance for Research in Singapore, Singapore, Singapore; 2 Civil and Environmental Engineering, Nanyang Technological University, Singapore, Singapore; 3 School of Environmental Science and Engineering, Shandong University, Jinan, China; 4 Graduate School of Public Health, Seoul National University, Seoul, South Korea; 5 Civil and Environmental Engineering, University of California, Berkeley, California, United States of America; Telethon Institute for Child Health Research, AUSTRALIA

## Abstract

**Introduction:**

Biological particles deposit on air handling system filters as they process air. This study reports and interprets abundance and diversity information regarding biomass accumulation on ordinarily used filters acquired from several locations in a university environment.

**Methods:**

DNA-based analysis was applied both to quantify (via DNA fluorometry and qPCR) and to characterize (via high-throughput sequencing) the microbial material on filters, which mainly processed recirculated indoor air. Results were interpreted in relation to building occupancy and ventilation system operational parameters.

**Results:**

Based on accumulated biomass, average DNA concentrations per AHU filter surface area across nine indoor locations after twelve weeks of filter use were in the respective ranges 1.1 to 41 ng per cm^2^ for total DNA, 0.02 to 3.3 ng per cm^2^ for bacterial DNA and 0.2 to 2.0 ng DNA per cm^2^ for fungal DNA. The most abundant genera detected on the AHU filter samples were *Clostridium*, *Streptophyta*, *Bacillus*, *Acinetobacter* and *Ktedonobacter* for bacteria and *Aspergillus*, *Cladosporium*, *Nigrospora*, *Rigidoporus* and *Lentinus* for fungi. Conditional indoor airborne DNA concentrations (median (range)) were estimated to be 13 (2.6–107) pg/m^3^ for total DNA, 0.4 (0.05–8.4) pg/m^3^ for bacterial DNA and 2.3 (1.0–5.1) pg/m^3^ for fungal DNA.

**Conclusion:**

Conditional airborne concentrations and the relative abundances of selected groups of genera correlate well with occupancy level. Bacterial DNA was found to be more responsive than fungal DNA to differences in occupancy level and indoor environmental conditions.

## Introduction

Current building operation design and practice were developed without detailed knowledge about how the accumulation of biological material occurs in various parts of mechanical ventilation systems. It is feasible that processes such as microbial colonization and metabolism contribute to adverse consequences for occupants, such as malodors, allergic reactions, asthma or sick building syndrome [1,2].

Office buildings in Singapore are commonly equipped with central ventilation and air conditioning systems. In tropical climates, such systems are referred to as providing “air conditioning and mechanical ventilation” (ACMV), because heating is not needed. In common international usage, such systems would be described as “heating, ventilating and air conditioning” (HVAC). In this study, because thermal conditioning is not central to our purposes, we will make reference to the “ventilation” or “air handling” system.

Air handling unit (AHU) filters are integrated into ventilation systems as the first line of defence, to protect downstream equipment and (sometimes) building occupants in indoor environments. The accumulation of particulate matter (PM) on these filters is inevitable. Appreciating the importance of ventilation system filters in processing both recirculated indoor air and outdoor air for building occupants, researchers have begun to analyse the biological composition of particulate matter collected on these filters. One of the earliest such studies involving AHU filters was performed in two shopping centres in Singapore [3]. In that study, the microbiota measured on filters was primarily bacterial, which could have originated from indoor sources. Additional studies of the nature and abundance of biological materials on ventilation system filters followed. Noris et al. [4] used culture-based methods to quantify biomass recovered from both indoor and HVAC filter dust, complemented by diversity analysis. Pigeot-Remy et al. [5] studied the survivability of certain bacteria on HVAC filters. Hoisington et al. [6] characterized bacterial assemblages on the filters collected from different retail stores in the US. Liu et al. [7] analysed culturable bacteria and fungi on AHU filters in offices across Beijing. Most recently, Haaland and Siegel [8] have suggested that analysis of accumulated material on AHU filters could serve a “quantitative filter forensics” function in building investigations.

Specific evidence remains limited regarding factors affecting the presence, abundance, and behavior of biological materials on AHU filters. It is important to study these factors to better understand the strengths and limitations of AHU filters as part of the mechanical ventilation system and to possibly gain insight into the levels and diversity of indoor bioaerosols in occupied spaces. No prior study provides both culture-independent quantitative measurements plus diversity analysis of biomass accumulated on AHU filters serving separate indoor spaces. To report such information for a cross-sectional sample of university buildings in Singapore is the purpose of this paper.

Here, we report baseline quantitative measurements and microbial composition of biological materials accumulated on used AHU filters from several locations in modern buildings in a tropical climate. We also aim to perform interpretive analysis on how indoor conditions affect the quantity and diversity of bioaccumulation on these filters. Measurements were made using culture-independent DNA-based analysis techniques. To satisfy the above research goals, we assessed the biomass concentration recovered from normally used ventilation-system filters. We investigated the influence of occupancy on bioaccumulation on these filters in different indoor environments and undertook high-throughput sequencing that targeted bacterial 16S rDNA and fungal internal transcribed spacer (ITS) sequences to analyse the microbial composition on the ventilation system filters.

## Materials and methods

### Indoor environments

Ventilation system filters from nine air handling units (AHU) at Nanyang Technological University (NTU), Singapore, were acquired for this study. The filter samples were collected with permission from the Facilities Management office in NTU and their partner for air conditioning services in Singapore. The nine indoor environments were three libraries (Library 1, Library 2 and Library 3), five university staff office environments (Office C, Office 1, Office 2, Office 3 and Office 4) and one classroom (Classroom). All filters were indoor secondary filters (pleated polyester, MERV 8 rating, where MERV denotes the “minimum efficiency reporting value”). As we intended to sample actual used AHU filters, the nine environments were carefully selected to minimize in-situ differences for relevant indoor comparisons. These AHUs used the same type of filter, operated during the same periods of time and were reasonably close to each other (all situated within one building complex).

In Singapore, owing to the consistently high latent heat load, central air conditioning systems generally operate with a very high proportion of recirculated air and a correspondingly small proportion of outside air. For the buildings studied here, the sampled air-handling filters processed air that was a blend of >90% recirculated air and <10% outdoor air. In accordance with Singapore regulations, filters in mechanical ventilation systems are replaced within six months of installation. The filters in this study were routinely replaced every three months. All filters analyzed in this study were collected at the end of the customary three-month period of service.

Several recent studies have documented that human occupants can act as major primary and secondary sources of indoor bioaerosols, through shedding and resuspension [4, 9–12]. To assess the influence of occupancy on the accumulation of biological material on air-handling system filters, we conducted population counts in each of the nine indoor environments studied. The total occupancy levels were estimated by means of an hourly count at each site via direct observation. For each of three weeks, these counts were undertaken each hour that the space was normally open, with the sampled weeks selected to span the three months of filter operation.

The operating hours of the AHUs varied according to the opening hours of the indoor spaces. The offices followed a fixed schedule with Office C and Office 1 operated on average 60 h/wk; the student and staff offices (Offices 2, 3 and 4) were open for an average of 91 h/wk. The classroom’s AHU operated on average 26 h/wk. The three libraries operated on different schedules with average durations of 73 h/wk, 56 h/wk and 73 h/wk for Library 1, Library 2 and Library 3 respectively. The total numbers of occupants observed each day were tallied and the weekly average value is reported as an estimated integral measure in units of person-hours per week, *N*. Note that each observation during the hourly counts of one occupant was estimated to contribute one person-hour to the cumulative weekly occupancy for that space.

In addition to occupancy, indoor environments were also characterized based on several major features, such as a description of general usage, activities observed during occupancy counts, interior furnishings, and size. We also measured total filter area (*A*_*f*_, m^2^) and the respective airflow speed (m/s) to estimate the volumetric supply air flow rate (*Q*, m^3^/s). Detailed descriptions of the air flow speed measurement methods, metadata describing the indoor environments, and the population counts are available in the Supporting Information (S1 File).

### Sampling, DNA extraction and quantification

The used filters were acquired during routine replacement. Samples were obtained by cutting small pieces (~2 × 5 cm) from the used filter on site with sterilized scissors immediately before the filter was removed from its mount for replacement. Each sampled filter piece was transferred into a sterile 50 mL falcon tube and transported to the lab on ice for DNA extraction. At least seven filter pieces of the same size were collected from each location to provide material for backup and replication. All unprocessed filters were stored at -20°C.

Each of the 10-cm^2^ filter pieces was placed into a 5 mL bead beating tube of MOBIO Power Water kit (MO BIO, Carlsbad, CA, USA) for DNA extraction. The DNA extraction steps followed the manufacturer’s protocol with some modifications to improve DNA yield [13]. Briefly, the modifications included vortexing for two minutes after the addition of preheated solution PW1 (the lysing agent), followed by water-bath sonication at 65°C for 30 minutes. After sonication, the samples were vortexed again for 5 minutes as recommended by MO BIO. The rest of the extraction steps followed the MO BIO original protocol. Extraction replicates (3 replicates for each sample) were separate 10-cm^2^ filter pieces that were cut from the same filter panel.

The total DNA in each sample was measured by a fluorescent dye dsDNA binding assay (Qubit 2.0, Invitrogen, Life Technologies, Carlsbad, CA, USA). The Qubit high sensitivity (HS) dsDNA kit was used for processing all samples.

Bacterial and fungal DNA levels were quantified by means of qPCR (Step One Plus, ABI, Life Technologies, Carlsbad, CA, USA) using Applied Biosystems Taqman master mix and a set of universal bacterial and fungal primers and probe. The primers for bacteria targeted the 338 to 805 region of 16S rRNA with the forward primer BAC338F (5’–ACTCCTACGGGAGGCAG–3’), the reverse primer BAC805R (5’–GACTACCAGGGTATCTAATC–3’), and the Taqman probe BAC516F (6FAM–TGCCAGCAGCCGCGGTAATAC–3’–BBQ). The primers used for fungi targeted the fungal 18S rRNA gene [14] with the forward primer FungiQuant-F (5’–GGRAAACTCACCAGGTCCAG–3’), the reverse primer FungiQuant-R (5’–GSWCTATCCCCAKCACGA–3’) and the Taqman probe FungiQuant-PrbLNA (6FAM–TGGTGCATGGCCGTT–3’–BBQ).

We established standard curves for bacteria and fungi with DNA extracts from *Escherichia coli* (ATCC 15597) and *Aspergillus fumigatus* (ATCC 26644), respectively. DNA was extracted from suspensions of both organisms in water and a dilution series (10^−1^, 10^−2^, 10^−3^, 10^−4^, 10^−5^) was prepared. The DNA concentrations of the suspensions were measured by both fluorometry (ng DNA/μL) and qPCR (Ct) to establish standard curves with which to relate DNA concentrations to Ct values. Final DNA concentrations are presented in terms of DNA mass per AHU filter surface area (ng or pg DNA/cm^2^).

As a negative control, clean (unused) AHU filters were briefly installed in each AHU and immediately extracted using the same protocol. The quantities of DNA from blank samples were below the detection limits of Qubit and no amplification was detected in the qPCR run with 35 thermal cycles, suggesting that there was no significant contribution of biomass from the maintenance company personnel during filter replacement, from the researchers during sampling and analysis of the filter pieces, or from the AHU filter materials themselves.

### DNA sequencing

Only five samples (Office C, Office 3, Office 4, Classroom and Library 3) met the minimum DNA abundance requirement for sequencing. Bacterial 16S rDNA and fungal ITS regions were targeted for DNA sequence analyses. The sequences of universal bacterial primers were 5’-CCTACGGGNBGCASCAG-3’ for the forward primer 341f and 5’-GACTACNVGGGTATCTAATCC-3’ for the reverse primer 805r [15]. The sequences of universal fungal primers were 5’-GCATCGATGAAGAACGCAGC-3’ for the forward primer ITS3 and 5’-TCCTCCGCTTATTGATATGC-3’ for the reverse primer ITS4 [16]. These primer sequences were attached with the adapter sequences for Illumina MiSeq. Index PCR was subsequently performed with the Nextera XT dual index barcode kit (Illumina, Hayward CA, USA). Amplicon purification was performed after each PCR with the AMPure XP beads (Beckman Coulter, Indianapolis IN, USA) to remove primers and dimers. The size of each purified amplicon was assessed using the Agilent BioAnalyser DNA1000 chip, and the concentration of each library was determined using the KAPA quantification kit (Kapa Biosystems, Inc., Wilmington MA, USA). The libraries were pooled in equimolar amounts before loading onto the sequencer. The pooled library was sequenced (2×250 bp) by means of the Illumina MiSeq with the nano v2 reagent.

The poly N tails were removed from the sequences by Trimmomatic version 0.33 [17]. The read 1 and 2 sequences were joined with a minimum allowed overlap of 10 bp in QIIME [18]. Sequences shorter than 100 bp were removed by the Galaxy Tool version 1.1 [19–20]. Additionally, chimeric sequences were removed in mothur v.1.25.0 [21] against the fungalITSdatabase database containing named fungal ITS sequences [22] for fungi, and against the reference Ribosomal Database Project (RDP) database [23] for bacteria.

For the fungal ITS sequences, taxonomic assignments were performed using BLASTN version 2.2.28+ [24] against the fungalITSdatabaseID containing named fungal ITS sequences [22] and classified using FHiTINGS [25]. Prior to diversity analyses, 43,769 sequences were subsampled from each library and binned into operational taxonomic units (OTUs) at 97% sequence identity using mothur version 1.25.0 [21]. The Shannon indices were calculated.

For the bacterial 16S rDNA sequences, taxonomic assignments were performed using the RDP Naïve Bayesian Classifier [23] with 0.8 as a confidence cutoff value. The Shannon indices were calculated based on 38,947 sequences clustered into 97% OTUs subsampled from each library. The unprocessed DNA sequences obtained from this study have been deposited in the Sequence Read Archives (SRA) of the National Centre for Biotechnology Information (NCBI) under these accession numbers: SAMN06131037, SAMN06131038, SAMN06131039, SAMN06131040, SAMN06131041, and SAMN06131042 for bacteria sequences; and SAMN06131043, SAMN06131044, SAMN06131045, SAMN06131046, and SAMN06131047 for fungi sequences.

In addition to microbial assemblage descriptions, correlations between occupant intensity in the indoor environments (described in a subsequent section) and the combined relative abundances of selected groups of human associated genera as well as Shannon diversity index were calculated to judge if these human-related genera would tend to be more abundant/diverse in spaces with higher occupancy. Spearman’s rank correlation coefficients were calculated and the correlations were tested for significance at *p* < 0.05.

### Airborne DNA concentration and emission factor estimation

Estimating the total DNA accumulation rate and its apportionment (total, bacterial and fungal) on the ventilation system filter (*Df*, in units of mass per week) was achieved by upscaling the amount of DNA extracted from the small filter area (*Ae*, m^2^) to the actual filter area (including pleats) in the AHUs (*Af*, m^2^). The DNA concentrations measured by Qubit fluorometry (ng/μL) and qPCR (pg/μL) were first converted to extracted DNA mass (*De*, mass units) based on the elution volume used during extractions. Total rates of accumulated DNA masses were then obtained from the following equation:

$$Df=\frac{De\;\times \;\frac{Af}{Ae}}{12}$$ (1)

The appearance of the factor 12 in the denominator of Eq (1) converts total DNA mass accumulated to the average weekly mass accumulation rate for the 12-week filter deployment periods. This conversion was applied to facilitate comparison of *Df* to the occupancy counts, which were also expressed on a rate basis, in units of person-hours per week.

The DNA accumulation rate on the ventilation system filter (*Df*) was normalized by the average quantity of air estimated to have passed through the filter during each week (*Vs*, m^3^/wk). The volumetric air-sampling rate was estimated by multiplying the determined volumetric air-flow rate (*Q*, m^3^/s) by its weekly cumulative operating duration (*To*, s/wk) for each environment (S1 File). Upon normalization, the resulting ratio (Eqs 2 and 3) represents the time-average airborne DNA concentration transferred to the filter and accumulating throughout the deployment period.

Vs=Q×To (2)

$$C=\;\frac{Df}{Vs}$$ (3)

In addition to determining effective time-averaged DNA concentrations, *C*, we also explored the occupant-induced contribution to DNA accumulation on the ventilation system filters. This analysis is based on two key premises: (a) that occupants are important contributors to the airborne DNA accumulating on ventilation system filters that process recirculated air; and (b) for a given category of indoor environment, the total effective occupancy-associated emission rate of DNA should scale with the occupancy level.

The exploration of the occupancy-induced contribution to DNA accumulation on the filter was achieved by regressing the inferred concentration, *C*, against a measure of occupancy normalized by ventilation rate, *Nd* (person-h per m^3^). Eq (4) was applied to determine the normalized occupancy as the ratio of the person-h per week of occupancy (*N*) to the airflow volume per week flowing through the AHU filter (*Vs*).

$$Nd=\;\frac{N}{Vs}$$ (4)

For all indoor environments, we regressed the inferred DNA concentration, *C*, against the normalized occupancy, *Nd*. The slope, *E* (DNA mass per person-h of occupancy) represents an estimate of the occupancy-associated effective emission factor for DNA that accumulates on the AHU filter. We applied this analysis for total DNA as well as for bacterial and fungal DNA.

Pearson correlation was applied to determine the statistical significance of the association between *C* and *Nd*. In this analysis, both Pearson correlation coefficient (*R*) and its significance were calculated. A correlation was considered significant when the Pearson correlation coefficient satisfied the required value for *p* < 0.05 at *n* = 9.

We also converted DNA mass values for *C* and *E* to number of cells for bacterial DNA and to number of spores equivalent (SE) for fungal DNA based on the conversion method of Qian et al. [9]. For bacterial DNA, the conversion took into account that *E*. *coli* (the standard that we used) has seven 16S RNA operon copies for each genome and that the average number of 16S RNA gene operon copies in a general bacterial genome is estimated to be four [26]. For fungal DNA, the conversion was done assuming that *A*. *fumigatus* has fifty-five operon copies of 18S rRNA gene per genome [27]. As the number of operon copies of rRNA is known to be highly variable in fungal genomes (even within the same species), one cannot determine an average number for that applies for general environmental fungi. Therefore, we converted our fungal DNA to *A*. *fumigatus* spores equivalent (SE). On average, 0.0027 pg and 0.030 pg of DNA [28] is equivalent to one bacterial cell and one fungal SE, respectively, for these assumptions and approximations. This conversion approach was performed to provide additional insight in interpreting the findings. Because of inherent uncertainties, caution is advised in extrapolation.

## Results and discussion

### DNA mass accumulated on AHU filters

Table 1 lists the concentration of extracted DNA mass per filter surface area for each of the AHU filters. The quantities of DNA per filter area spanned these respective ranges: 1.1 to 41 ng per cm^2^ for total DNA, 0.02 to 3.3 ng per cm^2^ for bacterial DNA, and 0.2 to 2.0 ng DNA per cm^2^ for fungal DNA. In addition to microbial (bacterial and fungal) DNA, the total DNA includes contributions from human, plants or animal cells that accumulated on the filter. On average, the measured microbial DNA contributed approximately 17% (1.5 out of 8.9 ng/cm^2^) of total DNA mass accumulated on the AHU filters. In the classroom and offices, the proportional contribution of fungi to total DNA was substantially larger than in the libraries. In contrast, the proportional contribution of bacterial DNA to total DNA was substantially larger in the libraries as compared to the classroom and offices.

**Table 1:** DNA abundance per AHU filter surface area for total, bacterial and fungal DNA from the nine indoor environments.

Indoor environment	Occupancy (N, person-h/wk)	Total DNA/filter area^a^ (ng/cm^2^)	Bacterial DNA/filter area^a^ (pg/cm^2^)	Estimated bacterial cell density^b^ (10^3^ cells/cm^2^)	Fungal DNA/filter area^a^ (pg/cm^2^)	Estimated fungal density^b^ (10^3^ SE/cm^2^)
Office C	190	1.1 ± 0.2	23 ± 2	8.5	422 ± 15	14
Office 1	979	3.3 ± 0.3	79 ± 6	29	1200 ± 90	40
Office 2	1518	5.9 ± 0.8	80 ± 14	30	951 ± 99	32
Office 3	1005	11.0 ± 0.5	171 ± 10	63	1764 ± 99	59
Office 4	1938	5.9 ± 0.9	165 ± 55	61	777 ± 85	26
Classroom	2736	1.5 ± 0.5	68 ± 14	25	193 ± 36	6.4
Library 1	946	4.5 ± 1.1	150 ± 20	56	857 ± 137	29
Library 2	639	5.8 ± 0.5	945 ± 33	350	797 ± 31	27
Library 3	7232	41.3 ± 3.1	3250 ± 280	1200	1988 ± 228	66

^a^ Average values of total, bacterial and fungal DNA abundance per AHU filter surface area ± standard deviation from 3 biological extraction replicates.

^b^ Estimated cell (bacteria) or spore equivalent (SE, fungus) abundance per filter surface area from each location; only the average value is displayed, rounded to two significant figures.

The estimated bacterial cell and fungal spore equivalent (SE) densities in Table 1 present analogous results to the DNA concentration per filter area. Across the nine sampled sites, estimated bacterial cell concentrations on AHU filters ranged from 8500 to 1.2 million cells/cm^2^; for fungi, the corresponding range was 6400 to 66,000 SE/cm^2^.

### Bacterial and fungal diversity analysis

#### Bacterial and fungal composition on used AHU filters

**Fig 1:**
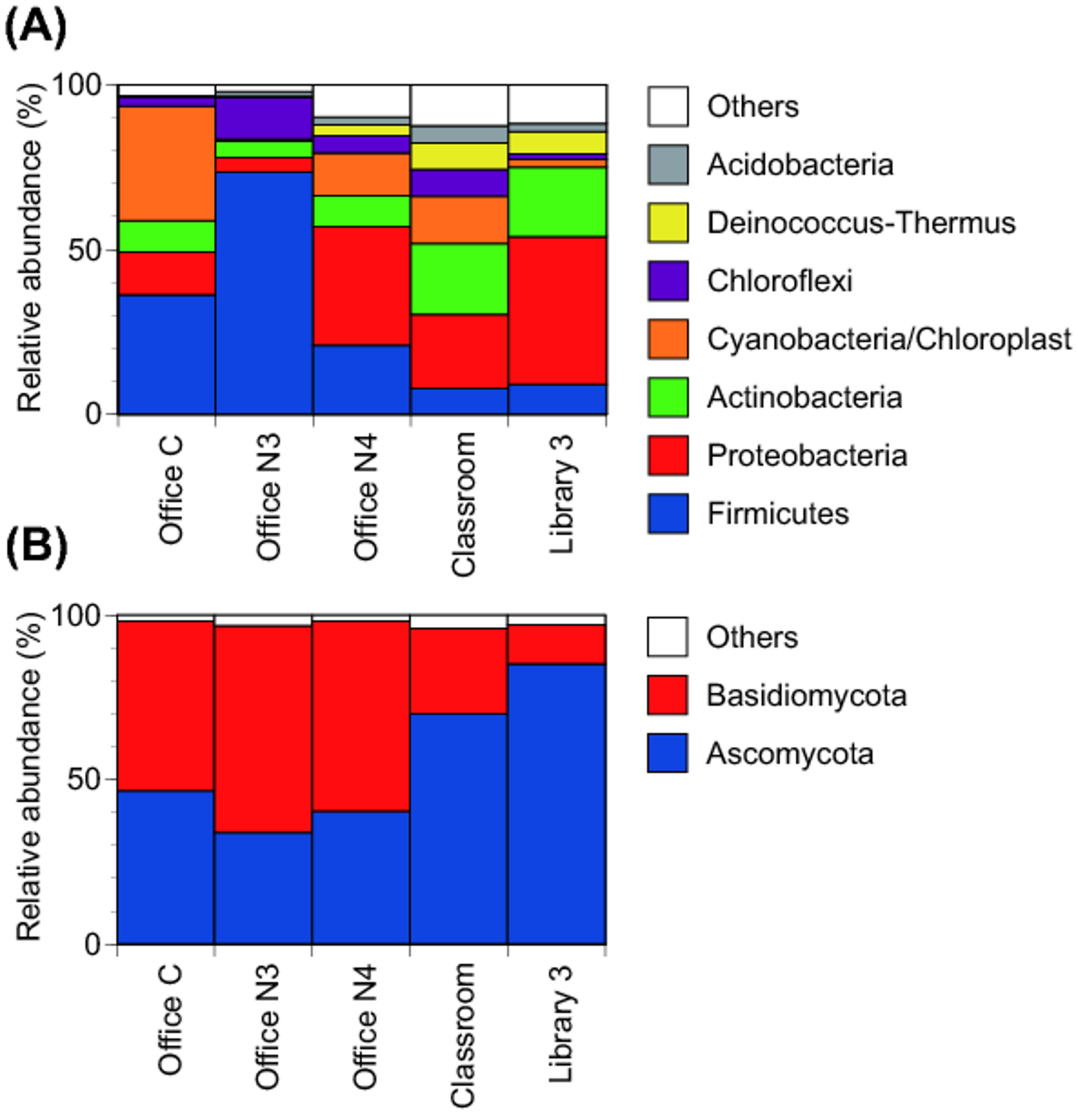
Bacterial and fungal phyla composition. Proportional distribution of bacterial (A) and fungal (B) phyla extracted from AHU filter samples from five locations.

**Fig 2:**
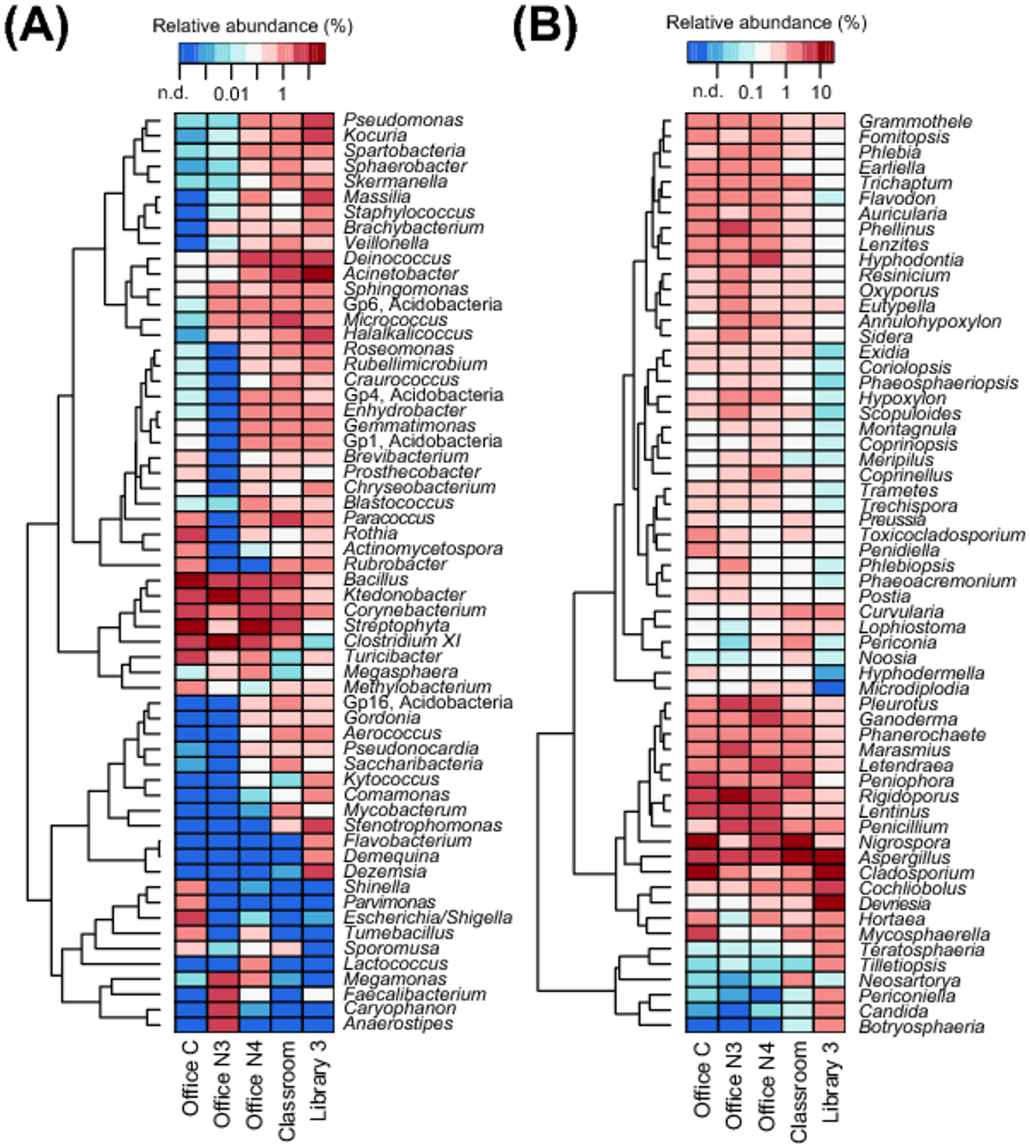
Bacterial and fungal genera heatmap. Relative abundances of the 40 most abundant bacterial (A) and fungal (B) genera. Taxa displayed represent 67% of all sequences for bacteria and 71% of all sequences for fungi.

In total, 385 bacterial and 783 fungal genera were detected from the five DNA samples that were sequenced (from Office C, Office 3, Office 4, Classroom and Library 3). Fig 1 displays the distributions of bacterial and fungal phyla. The three most abundant bacterial phyla were Firmicutes, Proteobacteria and Actinobacteria with average relative abundances of 30%, 24% and 13%, respectively. Firmicutes was detected more abundantly in the three offices, with relative abundances of 36% for Office C, 74% for Office 3, and 21% for Office 4; in the other locations, the relative abundances were 8% for the Classroom and 9% for Library 3. In contrast, Actinobacteria was more abundant in the Classroom (22%) and in Library 3 (21%) than in the offices: 10% for Office C, 5% for Office 3, and 10% for Office 4. Proteobacteria was most abundant in these three locations: 22% for the Classroom, 36% for Office 4 and 45% for Library 3. The five most abundant bacterial genera and their mean relative abundances were *Clostridium* (11%), *Streptophyta* (10%), *Bacillus* (6.7%), *Acinetobacter* (5.0%) and *Ktedonobacter* (3.6%). (See Fig 2(A).) Other prominent genera and their respective mean relative abundances were *Deinococcus* (3.5%), *Corynebacterium* (2.7%) and *Pseudomonas* (1.2%). To our knowledge, genera Gp1, Gp4, Gp6 and Gp16 have not been reported in previous indoor bioaerosol studies. These genera belong to phylum Acidobacteria, which are commonly found in soil or plant roots [29–30]. These bacteria likely originate outdoors. They could have reached the filter through the outdoor air intake or via human occupants as a secondary carrier. The dominant bacterial phyla and genera are generally in agreement with previous studies, which have analyzed bacterial assemblages on both ventilation system filters [3–4, 6, 10] and in indoor air [9–10, 31–32].

As illustrated in Fig 1(B), Basidiomycota was a dominant fungal phylum in the office environments, with relative abundances of 52% for Office C, 63% for Office 3, and 58% for Office 4. Ascomycota dominated the Classroom and Library 3 samples, with respective relative abundances of 70% and 85%. On average, Ascomycota had the higher average abundance (55%) than *Basidiomycota* (42%). The five most abundant fungal genera and their mean relative abundances were *Aspergillus* (11%), *Cladosporium* (7.4%), *Nigrospora* (4.9%), *Rigidoporus* (3.7%) and *Lentinus* (2.9%) (Fig 2(B)). Another prominent genus detected was *Penicillium* (2.2%). These findings generally conform to previous results, with *Aspergillus*, *Penicillium* and *Cladosporium* being abundant in both indoor and outdoor air across regions [33–35]. However, compared to bacteria, knowledge about fungal taxa on used AHU filters is relatively scarce. Our results are similar to those of Noris et al. [4], who reported fungal classes Dothideomycetes (contains genus *Cladosporium*), Sordariomycetes (contains genus *Nigrospora*) and Agaricomycetes (contains genera *Rigidoporus and Lentinus*) as the dominant fungi detected in filter dust samples.

Overall, the observed similarities substantiate the value of AHU filters in reflecting the microbial composition of the corresponding indoor air. More in-depth future studies on AHU filters are encouraged to better distinguish the specific causes of variability that has been reported among studies. For example, Proteobacteria in this study were more abundant in the locations with high occupancy (as described in subsequent sections), which seems contradictory to a previous observation that Proteobacteria on AHU filters are unlikely to be of human origin [4]. Fungal genera, such as *Alternaria* [34], *Cryptococcus* and *Epicoccum* [36] and bacterial genera, such as *Stenotrophomonas* [3] and *Methylobacterium* [6] were detected in much higher abundance in other studies as compared to this one.

#### Influence of occupancy on microbial diversity on AHU filters

While similarities were seen between our findings and previously reported investigations of indoor microbes, it is unknown whether correlations can be detected between the abundances of selected bacterial and fungal genera and potentially influential indoor conditions. Because the nature of the underlying distributions is unknown, we applied nonparametric Spearman’s rank tests. With a limited number of samples, the findings in this section are mainly intended to provide preliminary insight so as to guide the design of future studies that might use AHU filters as convenient samplers related to indoor environmental quality.

**Fig 3:**
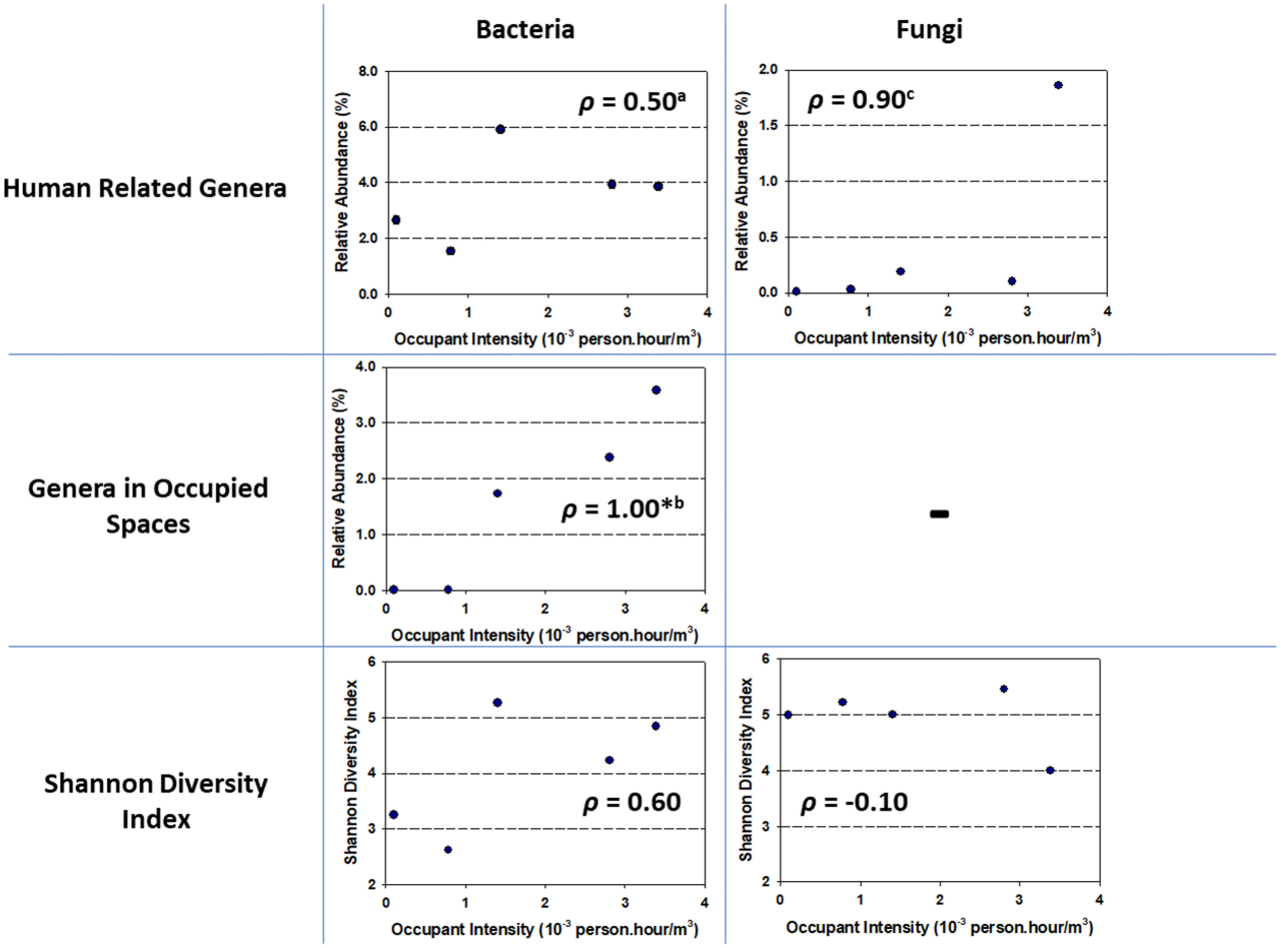
Spearman’s rank correlation between occupant intensity (*Nd*) and combined relative abundance of selected group of genera and Shannon diversity index values. The left frames showcase the correlations of bacteria and the right frames are for fungi. The symbol *ρ* indicates the Spearman’s rank correlation coefficient and * denotes statistical significance for the correlation at *p* < 0.05 with *n* = 5. ^a^ Correlation between occupant intensity and the group of human-related bacterial genera based on Qian et al. [9] and Hospodsky et al. [10]. ^b^ Correlation between occupant intensity and the group of bacterial genera commonly found in occupied spaces based on Kembel et al. [32]. ^c^ Correlation between occupant intensity and the group of human related fungal genera based on Yamamoto et al. [36].

Human-associated genera and genera known to be frequently detected in highly occupied spaces were selected to evaluate how their assemblages may vary on AHU filters in relation to human occupancy levels. Fig 3 showcases the Spearman’s rank coefficients (*ρ*) correlating occupant intensities (*Nd*) to relative abundances of selected groups of bacterial and fungal genera and to the α diversity index in the five locations. The group of human-related bacterial genera consists of *Corynebacterium*, *Propionibacterium*, *Enterobacter*, *Staphylococcus*, *Streptococcus*, *Fusobacterium* and *Veillonella* as skin or gut microflora [9–10]. A positive correlation (*ρ* = 0.5) was found between relative abundances of the group of human-related bacterial genera and human occupancy levels. Genera that are frequently detected in highly occupied spaces include *Lactococcus*, *Pseudomonas* and *Streptococcus* [32]. We also found a strong positive correlation between the relative abundances of this bacterial group and human occupancy levels (*ρ* = 1.00, *p* < 0.05). A positive correlation was also found between occupant intensity levels and the Shannon’s diversity index of bacterial assemblages (*ρ* = 0.60), indicating that the bacterial taxa on ventilation system filters tended to be more diverse with increasing occupancy levels. These findings regarding bacterial abundance and its correlation to occupancy are widely consistent with the understanding of human presence as a major source of bacterial emissions indoors.

For fungal taxa, a strong positive correlation (*ρ* = 0.90) was found between human occupancy levels and relative abundances of a group of human-related fungal genera of *Malassezia*, *Trichosporon*, *Rhodotorula*, *Cryptococcus*, *Pichia* and *Candida* [33]. The human related fungal genera in this study, however, only account for a much smaller portion of the total fungi (0.4%) as compared to fungal genera that are commonly found in both indoor or outdoor environments (20%), i.e., *Aspergillus*, *Penicillium* and *Cladosporium* [33–35, 37]. While some fungi deposited on AHU filters may originate from the outdoor air intake, occupants have also been reported to be either primary contributors [36] or secondary carriers [9] of indoor airborne fungi from various sources through several mechanisms such as resuspension from flooring and shoes, or shedding from clothes. Judging from the substantially higher combined abundance of environmental fungi compared to the combined abundance of human-related fungi, human occupants seem to mainly assume the roles of being secondary carriers or resuspending previously deposited environmental materials in contributing to the airborne fungi that deposited on AHU filters. No strong correlation was found between occupancy and the Shannon diversity index for fungal assemblages (*ρ* = -0.10). One possible explanation for this finding is because, as a secondary carrier, human occupants carry the same fungi from the outdoor environment and would not contribute to the diversity of accumulated fungi. Concurrently, as primary contributor, the small proportions of human related fungal genera relative to the total fungi on the filter are expected to have little influence on the Shannon index values, which account for both richness and evenness of a microbial assemblage.

#### Conditional airborne DNA concentrations and occupancy-related emission factors

Utilizing AHU filters as indoor air samplers is an attractive approach for indoor environmental quality studies. As shown by previous studies [3–4, 7], it is possible to consider an AHU filter as an integrating indoor air sampler, which can be acquired for quantitative analysis purposes with relatively little incremental effort. One of the benefits of utilizing AHU filters as samplers is the ability to overcome the relatively low limit of detection (LOD) of targeted airborne contaminants owing to the large sampling durations and high volumetric flow rates [8]. In practice, analyzing a building’s used ventilation system filters could also help to evaluate operational parameters, such as choice of filter grade or filter replacement frequency. In this section, we estimate airborne DNA concentration (*C*) based on AHU filter sampling. In addition, correlations between *C* and occupant intensity (*Nd*) are assessed as a basis for estimating occupancy-associated emission factors for indoor bioaerosols that accumulate on AHU filters (*E*).

Before presenting results, we would first state two cautions in interpreting *C* as the time-averaged indoor air concentration of DNA for the AHU’s operating hours. First, as indicated by Haaland and Siegel [8], an important uncertainty in quantitative filter forensics lies in determining actual filter efficiency. Filter efficiency varies with particle size, might be low for some filters, and can vary with time during the period of operation between replacements. Second, there is a concern that DNA may not be conserved on the filter. There has been evidence that bacteria could stay viable and remain active on ventilation system filters, instigating the possibility of replication [5]. Such activity could affect the microbial population dynamics on the filter surface over time. Conversely, a bioaerosol study utilizing filter membrane sampling method and another study investigating the time-series profile of DNA accumulation on AHU filters [13, 38] suggest that airborne DNA trapped on the filter may be lost during the relatively long filter deployment period (days for filter membrane and months for AHU filters). The studies showed that, despite continuous treatment of air, the cumulative DNA mass on the filter sometimes declined from one week to the next, possibly causing error in interpreting the quantitative results. Possible contributors to DNA loss include physical dislodging of particles during operation or DNA degradation on the filter surface owing to sampling-related stresses such as airflow induced shear or relatively high temperatures during periods of inoperation.

Although it is probable that the collection efficiency and DNA conservation of the filters analyzed were not 100%, for the quantitative comparisons made in this section, we make the approximation that collection efficiency is effectively 100% and that DNA collected on AHU filters is conserved. Two lines of reasoning support these assumptions. First, the target contaminants for all filters are the same: DNA that one expects to find mainly in coarse-mode particles, which can be captured with relatively high efficiency by MERV 8 filters. Second, to minimize in-situ and temporal variations, we carefully selected nine AHUs, which had the same filter type, operated during the same periods, and served rooms located in close proximity to one other. A few other cautions are noted that could also limit the accuracy of the interpretation. (a) Although most of the air passing through the filter is recirculated indoor air, there is a small contribution (< 10%) of outdoor air, so some of the accumulated DNA would have originated outdoors. (b) There may be transport losses that influence the relationship between room average concentrations and those in the return air passing through the AHU filter. Owing to all of these considerations, the reported airborne DNA concentrations and the occupancy-associated emission factors (*C* and *E*) should be considered as “conditional” findings, which are indicative of true circumstances, but which are also subject to uncertainties that cannot be quantified with available knowledge and information.

Table 2 presents the conditional airborne DNA concentrations (*C*) based on the analysis of ventilation-system filters. For total DNA, airborne concentrations in the nine locations varied between 2.6 pg/m^3^ and 107 pg/m^3^. There was a tendency for higher total DNA concentrations to be found in the libraries, with these sites exhibiting three of the highest four individual levels, the highest overall level, and an average (48 pg/m^3^) that was more than 4× the corresponding mean for offices and the classroom (11 pg/m^3^). Average airborne bacterial DNA concentrations were in the range 0.05–8.4 pg/m^3^ with an overall average across all indoor environments of 1.6 pg/m^3^. For fungal DNA, the concentrations span a narrower range, 1.0–5.1 pg/m^3^ and exhibit an average of 2.5 pg/m^3^. The bacterial and fungal DNA concentrations were also converted to bacterial cells and fungal spore equivalents to provide additional perspective. The time-averaged conditional airborne bacteria and fungi concentrations ranged from 20 to 3100 cells/m^3^ (average = 580) and from 33 to 173 SE/m^3^ (average = 83), respectively.

**Table 2:** Conditional airborne concentrations for total, bacterial and fungal DNA extracted from AHU filters serving nine indoor environments.

Indoor environment	Occupant intensity (*Nd*, person-h/m^3^)	Total DNA concentration^a^ (*C*, pg/m^3^)	Bacterial DNA concentration^a^ (*C*, pg/m^3^)	Estimated bacterial cell concentration^b^(cells/m^3^)	Fungal DNA concentration^a^ (*C*, pg/m^3^)	Estimated fungal spore concentration^b^ (SE/m^3^)
Office C	0.10 × 10^−3^	2.6 ± 0.4	0.05 ± 0.01	20	1.0 ± 0.1	33
Office 1	0.58 × 10^−3^	6.3 ± 0.6	0.15 ± 0.01	56	2.3 ± 0.2	77
Office 2	0.62 × 10^−3^	7.2 ± 1.0	0.10 ± 0.02	40	1.2 ± 0.1	40
Office 3	0.79 × 10^−3^	12.9 ± 0.6	0.20 ± 0.01	75	2.1 ± 0.1	70
Office 4	2.80 × 10^−3^	28.5 ± 4.2	0.80 ± 0.30	300	3.7 ± 0.4	120
Classroom	1.40 × 10^−3^	8.5 ± 3.1	0.40 ± 0.10	150	1.1 ± 0.2	36
Office + Classroom Avg^c^		11.0 ± 7.8	0.28 ± 0.26	110	1.9 ± 0.9	70
Library 1	0.50 × 10^−3^	14.5 ± 3.5	0.50 ± 0.10	190	2.8 ± 0.4	93
Library 2	1.20 × 10^−3^	21.6 ± 1.9	3.5 ± 0.1	1300	3.0 ± 0.1	100
Library 3	3.50 × 10^−3^	107 ± 8	8.4 ± 0.7	3100	5.1 ± 0.6	170
Library Avg^c^		48 ± 42	4.1 ± 3.3	1500	3.7 ± 1.1	120
Overall Average^c^		23 ± 31	1.6 ± 2.6	580	2.5 ± 1.3	83

^a^ Conditional DNA concentration: average ± standard deviation from biological replicates.

^b^ Conditional airborne cell (bacteria) and SE (fungus) concentration derived from AHU filter sampling; only the average value from each location is displayed.

^c^ Location-based averages calculated based on average values of each location ± standard deviation from the averages of each location.

**Fig 4:**
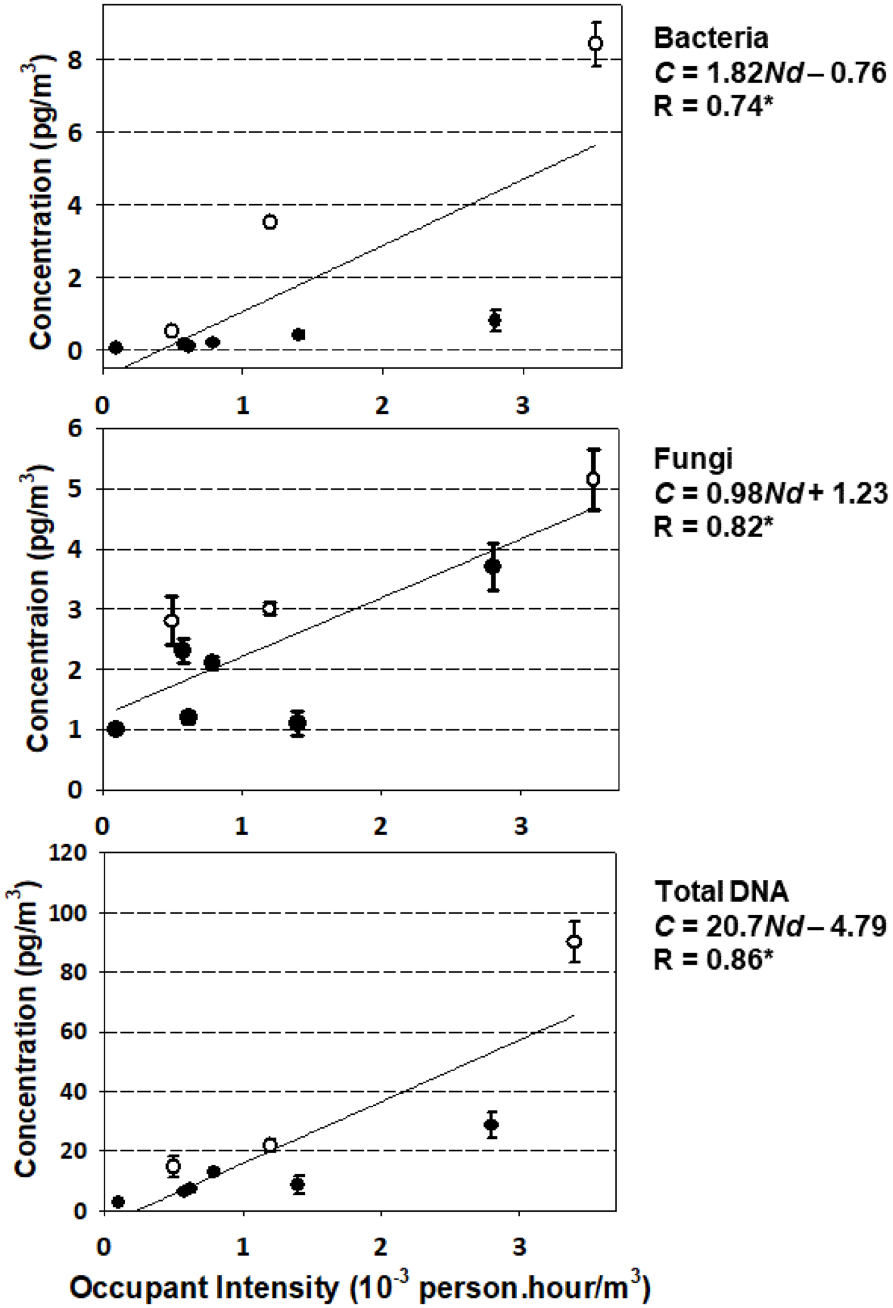
Correlations between airborne biomass concentration (*C*) and occupant intensity (*Nd*), for bacterial, fungal and total DNA. The white dots represent the libraries while the black dots represent the classroom and offices. The symbol *R* denotes the Pearson correlation coefficient. The symbol * indicates a statistically significant correlation at *p* < 0.05 with *n* = 9. The slope estimates a conditional emission factor of DNA associated with human occupancy.

Fig 4 illustrates the correlation between conditional airborne DNA concentrations (*C*) and the ventilation-rate-normalized occupancy level (*Nd*) for total, bacterial and fungal DNA. Overall, the values of Pearson correlation coefficients (*R*) indicate statistically significant moderate to high positive correlations across all three plots, indicating that the accumulation of the three categories of DNA are all positively related to this measure of occupancy in indoor locations.

The slopes of the three correlations can be used to estimate the occupant-associated DNA emission factor for bioaccumulation on AHU filters (*E*, ng DNA/(person-h)). As illustrated in Fig 4, the conditional DNA emission factors are 21 ng/person-h for total DNA, 1.8 ng/person-h for bacterial DNA, and 1.0 ng/person-h for fungal DNA.

Worth noting is the possibility for the two groups of locations (libraries and offices + classroom) to have different human associated emission factors. Based on the positions of the white and black dots in Fig 4, the possible differences are strongly suggested for total and bacterial DNA. If one were to separate the plots into two groups of locations for the three biological entities, the data would indicate systematically higher slopes and conditional emission factors for total DNA (3.5×) and bacterial DNA (8.6×) in the libraries as compared to the offices + classroom. On the contrary, fungal DNA emission factors would be similar in these two environment categories (less than 10% difference). Due to the strict criteria in AHU selection, however, we had a limited number of samples to confidently separate the plots into two groups of locations. We are highlighting this finding because it is supported by previous studies on the relations between human occupants and indoor bacteria [9–12]. These possible differences in emission factors would suggest that occupants shed and/or resuspend biomass at a more rapid rate, particularly in the case of bacteria, in locations with higher occupant activity levels. In the libraries, we observed that many occupants were actively walking with carts and old books, whereas occupants were mostly seated and working at desks in the offices and the classroom.

## Conclusions

This study is the first to provide baseline culture-independent abundance and diversity information on biomass that accumulated on AHU filters in several normally functioning office, classroom, and library environments in a university in a tropical climate. After 12 weeks of use, accumulated DNA masses per AHU filter surface area across nine indoor locations were determined to be in the respective ranges 1.1 to 41 ng/cm^2^ for total DNA, 0.02 to 3.3 ng/cm^2^ for bacterial DNA and 0.2 to 2.0 ng/cm^2^ for fungal DNA. The accumulation of all DNA types generally showed good correlation with occupancy level. Bacterial DNA was the most sensitive in relation to indoor occupancy.

In agreement with previous indoor biome studies, *Proteobacteria*, *Actinobacteria* and *Firmicutes* were the most abundant bacterial phyla detected and *Corynebacterium*, *Pseudomonas*, *Bacillus*, *Streptophyta* and *Acinetobacter* were among the most abundant bacterial genera. The most abundant fungal phylum detected on our AHU filters was *Ascomycota*, while the most abundant fungal genera were *Aspergillus*, *Cladosporium*, *Nigrospora*, *Rigidoporus* and *Lentinus*. Overall, changes in occupancy level were well reflected in the relative abundances of human-related bacterial and fungal genera.

In the context of indoor bioaerosol studies, our findings provide qualified support for the potential use of AHU filters as an indoor air quality probe. As recently noted by Haaland and Siegel [8], AHU filter sampling provides a convenient alternative to direct indoor air sampling for gathering enough particulate matter (including bioaerosols) from air for numerous analysis purposes. The correlation and microbial diversity analysis in this study showed how bioaccumulation on AHU filters corresponds well with changes in various indoor conditions, such as occupancy level or occupant activity intensity. There are, however, cautions to consider, such as filter efficiency differences and changes over time and also a potential lack of DNA conservation. These concerns need further study to improve understanding about the accuracy of AHU filter analysis for characterizing indoor bioaerosols. Future studies involving larger samples size, testing different filter types and grades, and a controlled effort to compare indoor air sampling to its corresponding AHU filter sampling are warranted to gain deeper understanding of these concerns and also to continue to advance knowledge regarding the roles of air handling systems in indoor bioaerosol dynamics.

## Supporting information

S1 File Indoor environment descriptions, air flow speed measurements, occupancy estimates, rarefaction curve, principal coordinate analysis and diversity indices. (XLSX)
